# Feasibility and User Experience of Digital Patient Monitoring for Real-World Patients With Lung or Breast Cancer

**DOI:** 10.1093/oncolo/oyad289

**Published:** 2023-11-25

**Authors:** Edurne Arriola, Jana Jaal, Anne Edvardsen, Maria Silvoniemi, António Araújo, Anders Vikström, Eleni Zairi, Mari Carmen Rodriguez-Mues, Marco Roccato, Sophie Schneider, Johannes Ammann

**Affiliations:** Medical Oncology Department, Hospital del Mar, Barcelona, Spain; Department of Hematology and Oncology, University of Tartu, Tartu, Estonia; Department of Pulmonary Medicine, Akershus University Hospital, Lørenskog, Norway; Department of Pulmonary Medicine, Turku University Hospital, Turku, Finland; Department of Medical Oncology, Centro Hospitalar Universitário de Santo António, Porto, Portugal; UMIB - Unit for Multidisciplinary Research in Biomedicine, ICBAS - School of Medicine and Biomedical Sciences, University of Porto, Porto, Portugal; Pulmonary Clinic, University Hospital, Linköping, Sweden; Medical Oncology Department, St. Luke’s Hospital, Thessaloniki, Greece; Medical Oncology Department, Hospital Clínico Barcelona, Barcelona, Spain; Program Manager Office (PMO), Kaiku Health, Helsinki, Finland; Pharma Personalised Healthcare, F. Hoffmann-La Roche Ltd, Basel, Switzerland; Global Product Development Medical Affairs, F. Hoffmann-La Roche Ltd, Basel, Switzerland

**Keywords:** digital patient monitoring, patient-reported outcomes, lung cancer, breast cancer, symptom monitoring, eHealth

## Abstract

**Background:**

Digital patient monitoring (DPM) tools can facilitate early symptom management for patients with cancer through systematic symptom reporting; however, low adherence can be a challenge. We assessed patient/healthcare professional (HCP) use of DPM in routine clinical practice.

**Materials and Methods:**

Patients with locally advanced/metastatic lung cancer or HER2-positive breast cancer received locally approved/reimbursed drugs alongside DPM, with elements tailored by F. Hoffmann-La Roche Ltd, on the Kaiku Health DPM platform. Patient access to the DPM tool was through their own devices (eg, laptops, PCs, smartphones, or tablets), via either a browser or an app on Apple iOS or Android devices. Coprimary endpoints were patient DPM tool adoption (positive threshold: 60%) and week 1-6 adherence to weekly symptom reporting (positive threshold: 70%). Secondary endpoints included experience and clinical impact.

**Results:**

At data cutoff (June 9, 2022), adoption was 85% and adherence was 76%. Customer satisfaction and effort scores for patients were 76% and 82%, respectively, and 83% and 79% for HCPs. Patients spent approximately 10 minutes using the DPM tool and completed approximately 1.0 symptom questionnaires per week (completion time 1-4 minutes). HCPs spent approximately 1-3 minutes a week using the tool per patient. Median time to HCP review for alerted versus non-alerted symptom questionnaires was 19.6 versus 21.5 hours. Most patients and HCPs felt that the DPM tool covered/mostly covered symptoms experienced (71% and 75%), was educational (65% and 92%), and improved patient-HCP conversations (70% and 83%) and cancer care (51% and 71%).

**Conclusion:**

The DPM tool demonstrated positive adoption, adherence, and user experience for patients with lung/breast cancer, suggesting that DPM tools may benefit clinical cancer care.

## Teaser

Digital patient monitoring tools can facilitate early symptom monitoring and management for cancer through systematic symptom reporting; however, low adherence can be a challenge. This study assessed patient and healthcare professional use of such tools in routine clinical practice.

**Implications for Practice**

Adherence to digital health solutions over time is a challenge. We conducted a real-world study to assess patient adoption and adherence, and patient and healthcare professional (HCP) user experience of digital patient monitoring (DPM) for patients with lung/breast cancer in routine clinical practice. Our results showed that patient adoption of the DPM tool and adherence to weekly symptom reporting was high, and that most patients and HCPs had a positive user and care impact experience. This suggests that our DPM tool may facilitate symptom detection in outpatient clinics and improve early management and clinical cancer care.

## Introduction

Poorly controlled disease- and treatment-related symptoms can impact negatively on clinical outcomes and quality of life (QoL) in patients with cancer^1-3^; eg, nausea and vomiting, the most frequently reported symptoms from chemotherapy, can significantly affect patients’ daily lives and consequently can lead to treatment discontinuation.^4,5^ Early symptom management can improve QoL, depression, mood, and overall survival versus standard care.^6,7^ However, patients typically only report symptoms during scheduled clinic visits. This may affect accuracy and details of reporting due to recall bias and difficulties in memorizing symptoms, challenges in the healthcare professional (HCP)-patient relationship, and the patient’s emotional state.^8-10^ In addition, the rising incidence and prevalence of cancer coupled with shortages of HCPs and longer intervals between hospital visits, potentially leading to reduced/less frequent HCP-patient contact time, may result in fewer symptoms being reported.^11-14^

Digital patient monitoring (DPM; remote patient monitoring) tools that enable patients with cancer to self-report disease-/treatment-related symptoms and QoL in a structured way, alert HCPs about critical symptoms, communicate directly with HCPs, and access self-management and support materials, have been investigated in clinical trials and introduced into clinical practice.^15,16^ The broad introduction of DPM tools into practice across different cancer indications and treatment lines is now recommended in the ESMO Clinical Practice Guideline.^17^ Properly implemented, these types of DPM tools can support improved patient-centered care by enabling higher congruence between symptoms being reported and addressed during clinic visits.^18^ DPM tools have also been shown to improve overall survival, QoL, treatment duration, and psychosocial outcomes, and to reduce symptom burden, including rates of severe and/or serious adverse events (AEs), the need for dose changes, and healthcare resource utilization by reducing emergency room visits and hospitalizations.^19-36^ Furthermore, some reports have suggested that they could facilitate risk prediction for future AEs.^37-39^

Studies of eHealth tools have shown challenges from low adherence over time.^40^ For DPM tools to be successfully integrated into practice and used in evidence generation, it is important to understand HCP and patient adoption, adherence, and user experience. We describe results from a real-world evidence study designed to assess adoption, adherence, and user experience of a DPM tool with tailored content for patients with lung/breast cancer in routine clinical practice. The content was tailored by F. Hoffmann-La Roche Ltd (Basel, Switzerland) based on learnings from a previous pilot study, and hosted on the Kaiku Health (Helsinki, Finland) DPM platform.^27^ Selected clinics were provided with access to the Kaiku Health DPM platform, which is a Class IIa active medical device both under the Medical Devices Directive according to Rule 10 (current at time of writing) and under the Medical Devices Regulation according to Rule 11.^41,42^ Patient access to the DPM tool was through their own devices (eg, laptops, PCs, smartphones, or tablets), either via a browser or via an app on Apple iOS or Android devices. HCPs (providers from routine practice) accessed the tool with the appropriate hospital devices used in their routine work. Patients could use the tool to report their health and wellbeing using predefined disease- and treatment-specific symptom/health-related QoL (HRQoL) questionnaires in DPM modules; view a dashboard of reported symptoms; receive reminders, notifications, and instructions related to reporting of predefined metrics; access patient materials such as self-management support instructions for selected mild/moderate symptoms^43-46^; and send messages and attachments to their care team. Relevant symptoms for the symptom questionnaires were selected based on the drug’s/drug class’s AE profile of the disease’s symptoms. The selection was refined in a discussion process with physicians, nurses, and patient groups. Symptoms covered by the DPM tool are listed in Supplementary Table S1. HCPs could use the tool to assign patients to predefined and individualized modules; receive, input, and view individual patient symptom reports; access an automated triage view of incoming symptom reports; and send messages and attachments to patients.

## Methods

### Recruitment and Participants

HCPs and patients were recruited from 10 clinics across Estonia, Finland, Greece, Norway, Portugal, Spain, and Sweden between March 2021 and June 2022. Eligibility criteria are shown in the Supplementary Materials.

### The DPM Tool

Patients were prompted weekly to complete a symptom questionnaire in the DPM tool or could report their symptoms on an ad hoc basis. HCPs received daily digest emails about new patient reports; if a patient reported a new grade 3 symptom or ≥2-grade change in any symptom, an additional email notification was sent within 15 minutes of the patient’s report. Notifications of symptom reports were sent to HCPs in order to prioritize review and management, based on a composite grading approach.^47^ It was recommended to assign an HCP per site to monitor the dashboard daily on workdays. Patients and HCPs used the DPM tool for up to 15 months for symptom reporting and management; HRQoL data were collected up to week 18 (based on median duration of first-line cancer immunotherapy treatment in advanced non-small cell lung cancer and small cell lung cancer/first-line HER2-targeted treatment in advanced breast cancer).

### Data Collection and Analysis

The coprimary endpoints were patient adoption of the DPM tool (percentage of patients who accepted the invitation from their HCP to use the tool) and adherence to weekly symptom self-reporting in weeks 1-6. Patient adoption was considered positive if ≥60%, based on previous studies.^36,48-52^ Data on the number of patients adopting DPM were extracted from the DPM tool. HCPs completed a questionnaire for every patient who was offered the tool but declined. Adherence was considered positive if ≥70% patients (based on previous studies^48,50-53^) logged in to the tool weekly in weeks 1-6 of use. Data were collected from the tool, and patients who dropped out of the study for reasons unrelated to the tool or who did not complete a symptom questionnaire for >4 weeks were excluded from the analysis. Other collected adherence data per patient were adherence over complete use time; duration of use in weeks; time spent using the tool, completing the symptom questionnaire, and using self-management or educational materials per week; number of logins, questionnaires completed, and messages between HCPs and patients per week; and time spent by HCPs on the tool per patient per week.

Secondary endpoints included perceived DPM tool user experience, clinical impact, patient communication and self-efficacy, and HRQoL. HCP and patient user experience were assessed via tool experience questionnaires at week 6. A net promoter score (NPS) >11 was considered a positive user experience for HCPs and patients, based on the pilot study.^27^ Clinical impact in terms of how the DPM tool helped HCPs and patients to become better informed, have better conversations, and improve care, as well as the estimated symptom coverage of the tool, was measured using an HCP care impact questionnaire and patient experience questionnaire at week 6.

Data on patient and HCP DPM user characteristics were collected using questionnaires at week 0.

Methods for collecting and analyzing symptom alert and HRQoL data are shown in the Supplementary Materials.

### Ethical Considerations

This research was conducted in full conformance with the International Society of Pharmacoepidemiology Guidelines for Good Pharmacoepidemiology Practice and the laws and regulations of the country in which the research was conducted. Depending on local regulations, the research plan and relevant supporting information were approved by the Institutional Review Board/Ethics Committee at the participating site. Patient data were anonymized. All participants provided written informed electronic consent upon logging in to the DPM tool for the first time.

## Results

### Patient Adoption of the DPM Tool and Adherence to Weekly Symptom Reporting

**Figure 1.**
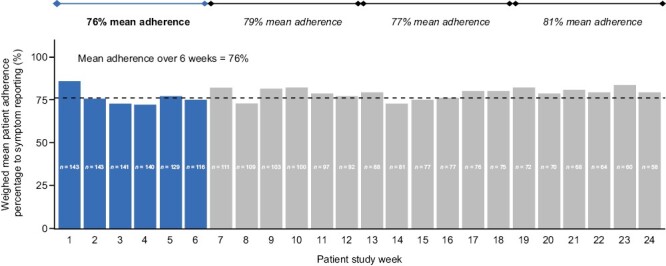
Patient adherence to symptom self-reporting over time. Patient numbers (*n*) represent the number of patients who were expected to complete a symptom questionnaire in the respective study week.

At data cutoff (June 9, 2022), 153 patients were enrolled (mean: 15 per clinic) and had provided data for up to 15 months. Eighty-nine patients used Roche-created DPM modules, with the remaining 64 using generic Kaiku-created modules. The DPM tool was adopted by 85% of patients. Mean patient adherence to weekly symptom reporting in the first 6 weeks was 76%; in subsequent 6-weekly intervals, mean adherence ranged between 77% and 81% (Fig. 1). Both adoption and adherence were above the threshold for positive endpoints (60% and 70%, respectively); therefore, the study met its coprimary endpoints.

### Characteristics of Questionnaire Respondents

Of the 153 patients enrolled, 146 responded to a questionnaire regarding patient characteristics; 96 (66%) had stage IV cancer. The proportion with an Eastern Cooperative Oncology Group (ECOG) performance status of 0, 1, 2, and 3 was 39% (*n* = 57), 51% (*n* = 75), 8% (*n* = 11), and 2% (*n* = 3), respectively.

**Figure 2.**
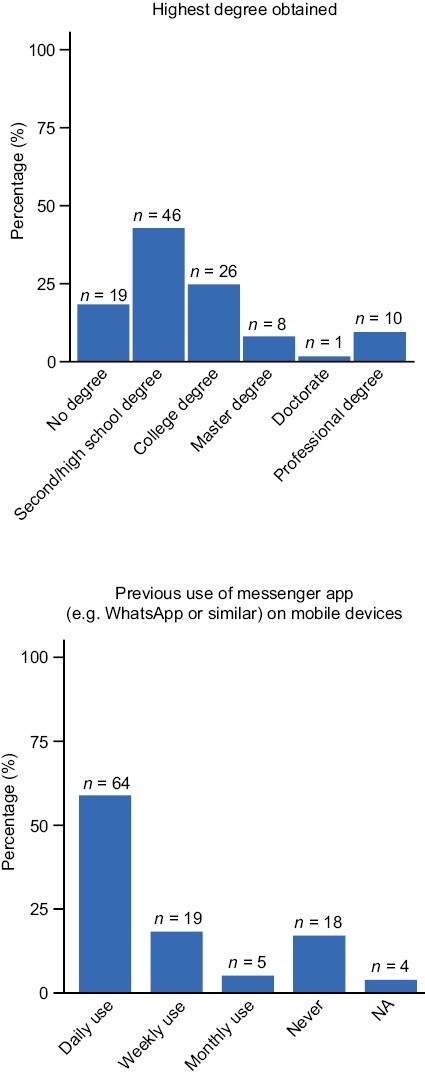
Patient education and previous messenger app usage. Abbreviation: NA: not applicable.

A total of 110 patients completed the DPM tool experience questionnaire at week 6. Of these respondents, 61 (55%) were male, and 91 (83%) had at least a secondary/high school degree (Fig. 2). Most patients (68 [62%]) were 60 years of age or over; 26 (24%) were 50-59, 11 (10%) were 40-49, and 5 (5%) were 30-39. Most of the patient users (106 [96%]) owned a tablet/mobile phone with internet connection, with 64 (58%) and 19 (17%) using messenger apps (eg, WhatsApp or similar) daily and weekly, respectively (Fig. 2), and 43 (39%) having used a similar DPM tool previously.

Of the 70 HCPs enrolled, 30 completed the HCP characteristics and onboarding experience questionnaire at week 0. Eleven (37%) were between 40 and 49 years of age, 9 (30%) were <40, and 10 (33%) were ≥50; 8 (27%) had previous experience with DPM tools. Sixty-seven percent of HCPs indicated that a physician/nurse had been assigned to monitor the dashboard on a daily basis. In 60% of these cases, it was a nurse (Supplementary Fig. S1).

### User Experience

**Figure 3.**
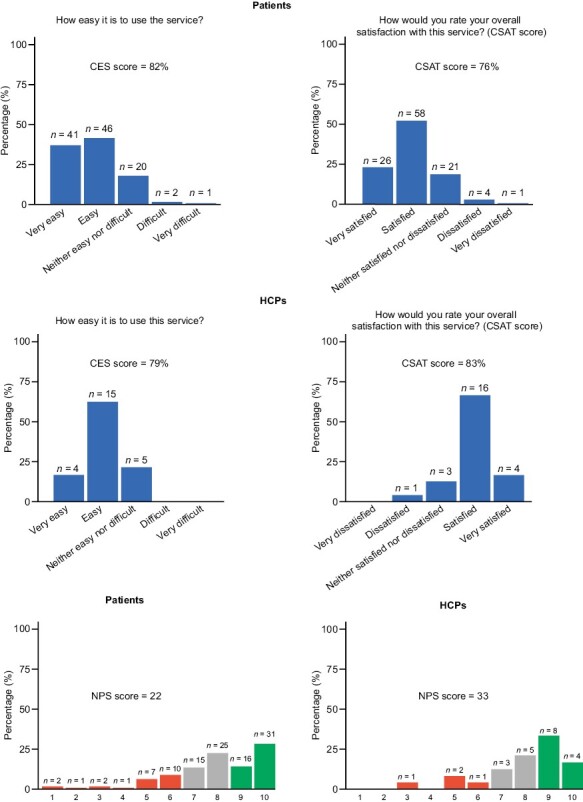
Patient and HCP user experience. Abbreviations: CES: customer effort score; CSAT: customer satisfaction score; HCP: healthcare professional; NPS: net promoter score.

In addition to the 110 patients who provided data on user experience, 24 HCPs also completed a DPM tool experience questionnaire at week 6. Most patients and HCPs were satisfied/very satisfied with the tool, with customer satisfaction (CSAT) scores of 76% and 83%, respectively (Fig. 3). They also found the tool easy/very easy to use (customer effort scores [CES] were 82% and 79%, respectively) (Fig. 3). Both CSAT and CES scores were higher for doctors versus nurses (CSAT: 100% vs. 75%; CES: 83% vs. 78%). NPS scores for patients and HCPs were positive (22 and 33, respectively) (Fig. 3). Further investigation of NPS scores also showed a difference between nurses and doctors (19 vs. 62, respectively).

HCPs received ~1 hour of online eTraining for the DPM tool. Most HCPs agreed/strongly agreed that this was well structured and easy to follow (*n* = 28/30 [93%]), that they were satisfied with the overall onboarding experience (29/30 [97%]), that they felt confident about using the tool (24/30 [80%]), and that they knew where to find instructions and/or whom to contact for help/advice on using the tool (28/30 [93%]).

### Clinical Practice Use

Patients spent a median of 10 minutes (range 5-33) per week logged in to the DPM tool across all modules. The median number of symptom questionnaires completed by patients per module was ~1.0 per week, each of which took a median of 1-4 minutes to complete. The median number of symptom questionnaires completed per week was similar across ECOG performance status and patient age groups. The median time to complete symptom questionnaires was higher in the group of patients ≥60 years versus 50-59 years, and in those with an ECOG performance status of 1 or 2 versus 0.

HCPs spent an average of 1-3 minutes per patient on the tool each week. For most clinics, nurses were the primary users of the DPM tool. The median time to HCP review for non-alerted symptom questionnaires was 21.5 hours; alerted symptom questionnaires, which accounted for ~25% of all questionnaires, were reviewed by HCPs in a median of 19.6 hours (Supplementary Fig. S2).

### Perceived Clinical Impact

**Figure 4.**
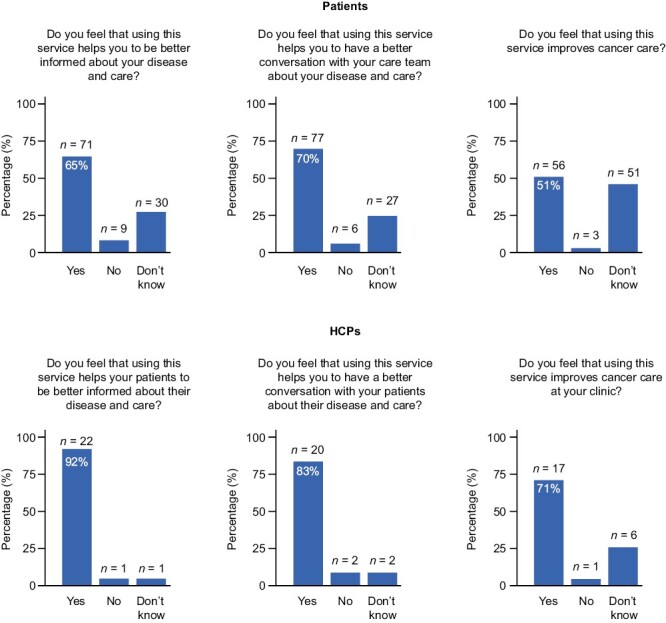
Perceived clinical impact of the DPM tool by HCPs and patients after 6 weeks of use. Abbreviations: DPM: digital patient monitoring; HCP: healthcare professional.

After 6 weeks of use, the majority of patients and HCPs felt that using the DPM tool helped them to be better informed about the disease and patient care (*n* = 71/110 [65%] and 22/24 [92%], respectively); improved conversations between patients and their care team (77/110 [70%] and 20/24 [83%]); and improved cancer care (56/110 [51%] and 17/24 [71%]) (Fig. 4).

Overall, 75% of HCPs and 71% of patients considered the symptom questionnaires to fully/mostly cover the symptoms experienced during the first 6 weeks of DPM use. A total of 47% of patients who reported their symptoms were not fully covered contacted their HCP team about such symptoms. HCPs mostly learned about symptoms not covered by the questionnaire via phone calls, during clinic visits, or via messages in the DPM tool.

### Impact on HRQoL

Exploratory HRQoL analyses of data from 45 patients who answered the questionnaire at baseline and weeks 6, 12, and 18 using the Wilcoxon signed rank test for non-normally distributed data demonstrated that the HRQoL summary score was significantly improved at weeks 12 (*P* = .046) and 18 (*P* = .042) versus baseline. They also showed that the HRQoL global health status and symptom control score were significantly improved at week 12 (*P* = .01 and *P* = .025, respectively) versus baseline, but not at week 6 (*P* = .52 and *P* = .23, respectively) or week 18 (*P* = .16 and *P* = .079, respectively) (Supplementary Fig. 3). Lower numbers for individual symptom burden were reported over time, but this was mostly not statistically significant due to limited data.

## Discussion

In this real-world study of a DPM tool for patients with lung/breast cancer, the coprimary endpoints of patient adoption of the DPM tool and adherence to symptom self-reporting at week 6 were met, confirming that the tool is an acceptable and desirable solution for patients. Our DPM tool’s adoption rate observed in this study (85%) is slightly higher than that observed in previous publications for similar DPM tools, where the reported adoption rates were in the range of 60%-75%.^36,48-52^ The observed adherence in this study (76%-81%), however, is within the range of weekly adherence rates from previous studies of the Patient-Reported Outcomes version of the Common Terminology Criteria for Adverse Events (PRO-CTCAE) reporting of similar DPM tools (70%-85%).^48,50-53^ One consideration that could potentially affect adherence is the frequency of the symptom questionnaires. In our study, patients were prompted to complete a questionnaire weekly. Changing the frequency of prompts to once every other week/once per month could improve adherence in patients who do not want to answer the same questions every week; however, this may increase the risk that some symptoms might not be reported.^8^ In addition, the DPM tool assessed in this study included modules that were disease- and treatment-specific. Treatment-specific DPM tools (eg, those for immunotherapy/targeted therapy for multiple cancer types) may be considered more useful than disease-specific DPM tools (eg, those only for breast cancer) in clinical practice as they allow use of the tool across different cancer sites. This could also further improve adoption and adherence of DPM tools.

The user experience of the DPM tool was positive for both patients and HCPs, which was confirmed by the high CSAT, CES, and NPS scores. As the time point of questionnaire administration was the same for all patients (after 6 weeks of DMP tool use) and the questions focused on the satisfaction with the DPM service/tool, we do not expect an impact from the start of drug treatment. However, nurses had consistently lower scores than doctors, which could be indicative of the increased workload for nurses caring for patients using the DPM tool, as nurses were the primary users of the tool at most of the participating sites. Alternatively, the poor integration between the DPM tool and electronic health records may have caused lower scores from nurses who are responsible for maintaining patient records. On the other hand, doctors may have been more committed to using the DPM tool, with more awareness of the difficulties of patient-doctor communication in standard clinical practice.

Time spent using the tool for patients and for HCPs per patient each week was low, as was the time required to complete a symptom questionnaire. The time measurements obtained in this study suggest that the burden on patients and HCPs of using the DPM tool is low. In addition, there was no significant difference between the numbers of questionnaires completed by patients across all ECOG performance status and age groups, which indicates that the DPM tool is suitable for a broad patient population, although the time to complete a symptom questionnaire was slightly higher in older patients and patients with worse performance status.

The small time difference between alerted and non-alerted symptom reviews by HCPs (21.5 vs. 19.6 hours) is likely due to the fact that most clinics performed triage each morning and that patients with new symptoms would often contact the clinic themselves rather than waiting for HCP review of the DPM alerts, per the usual care construct. Consistent with previous reports,^54^ this suggests that alerting critical symptoms to HCPs versus patients does not impact the utility of a DPM tool.

Most patients and HCPs agreed that the DPM tool had a positive clinical impact by helping them to be better informed about the disease and care, to have better conversations between patients and their care team, and to improve cancer care. Exploratory analyses also demonstrated a benefit in terms of HRQoL, with the DPM tool resulting in significantly improved HRQoL summary scores at weeks 12 and 18 versus baseline, and significantly improved HRQoL global health status and symptom control score at week 12 versus baseline. Improvements in HRQoL scores with DPM use in this study are consistent with other studies demonstrating an increase in QoL in patients using DPM tools based on the EQ-5D-5L, the Functional Assessment of Cancer Therapy (FACT), or the QLQ-C30 questionnaire instruments, versus no DPM tool use.^19,21,23,55-57^ To better describe the impact of our DPM tool on HRQoL over time, we have recently begun a clinical trial in which patients will be randomized into a DPM arm and a non-DPM “control” arm.^58^

The analyses presented here have several strengths. Being a real-world study, the data collected are reflective of adoption, adherence, and user experience in routine clinical practice in populations of patients with different cancer diagnoses and treatments. In addition, the usage data were collected from the tool’s analytics wherever possible, both to reduce the number of questions to be answered by HCPs and patients and to circumvent perception biases.

Study limitations include the possibility of incomplete data collection via questionnaires with a likely decrease in questionnaire respondents and completeness over time as shown in similar studies,^53,55^ due to the nature of the project setting and patient population and the potential bias on adoption rates caused by preselection of patients by HCPs. Furthermore, our study lacked a control group and included low sample sizes, which in particular limits the interpretation of HRQoL results (<1/3 patients completed HRQoL questionnaires at all timepoints). Finally, our study did not formally assess the impact of the DPM modules on patient outcomes, eg, overall survival, progression-free survival, or AEs; however, this will be the focus of a future randomized controlled trial (NCT05694013) by F. Hoffmann-La Roche Ltd, which is currently being conducted.

## Conclusion

Overall, our results demonstrated positive adoption, adherence, and user experience for patients with lung or breast cancer using a DPM tool alongside locally approved and reimbursed drugs. These results suggest that adoption of DPM tools into routine clinical practice is both feasible and can have a positive effect on clinical cancer care.

## Supplementary Material

Supplementary material is available at *The Oncologist* online.

oyad289_suppl_Supplementary_Material

## Data Availability

For up-to-date details on Roche’s Global Policy on the Sharing of Clinical Information and how to request access to related clinical study documents, see here: https://go.roche.com/data_sharing. Pseudonymized, individual patient level data are made available to qualified researchers by request from the corresponding author (johannes.ammann@roche.com). Anonymized records for individual patients across more than one data source external to Roche cannot, and should not, be linked due to a potential increase in risk of patient reidentification.
