# Supplementary material for: Feasibility and User Experience of Digital Patient Monitoring for Real-World Patients With Lung or Breast Cancer
Source: Oncologist. 2023 Nov 25;29(4):e561–9. doi: 10.1093/oncolo/oyad289 (PMC10994260; doi:10.1093/oncolo/oyad289)
Supplement: oyad289_suppl_Supplementary_Material [file oyad289_suppl_supplementary_material.docx]

**Feasibility and User Experience of Digital Patient Monitoring for Real-World Patients with** **Lung or Breast Cancer**

Edurne Arriola^1^, Jana Jaal^2^, Anne Edvardsen^3^, Maria Silvoniemi^4^, António Araújo^5,6^,
Anders Vikström^7^, Eleni Zairi^8^, Mari Carmen Rodriguez-Mues^9^, Marco Roccato^10^,
Sophie Schneider^11^, Johannes Ammann^12^*

**Author affiliations:**

^1^Medical Oncology Department, Hospital del Mar, Barcelona, Spain

^2^Department of Hematology and Oncology, University of Tartu, Tartu, Estonia

^3^Department of Pulmonary Medicine, Akershus University Hospital, Lørenskog, Norway

^4^Department of Pulmonary Medicine, Turku University Hospital, Turku, Finland

^5^Department of Medical Oncology, Centro Hospitalar Universitário de Santo António, Porto, Portugal

^6^UMIB - Unit for Multidisciplinary Research in Biomedicine, ICBAS - School of Medicine and Biomedical Sciences, University of Porto, Porto, Portugal

^7^Pulmonary Clinic, University Hospital, Linköping, Sweden

^8^Medical Oncology Department, St. Luke’s Hospital, Thessaloniki, Greece

^9^Medical Oncology Department, Hospital Clínico Barcelona, Barcelona, Spain

^10^Program Manager Office (PMO), Kaiku Health, Helsinki, Finland

^11^Pharma Personalised Healthcare, F. Hoffmann-La Roche Ltd, Basel, Switzerland

^12^Global Product Development Medical Affairs, F. Hoffmann-La Roche Ltd, Basel, Switzerland

*Corresponding author: Johannes Ammann, PhD, F. Hoffmann-La Roche Ltd, Grenzacherstrasse 124, 4070 Basel, Switzerland. Tel: +41 792 921 758;
Email: johannes.ammann@roche.com

**Supplementary Online Methods**

**Eligibility**

Eligible patients were ≥18 years of age, had locally advanced or metastatic lung cancer (non-small cell lung cancer or small cell lung cancer) or HER2-positive breast cancer, were already on a drug treatment or starting a new drug treatment with locally approved and reimbursed drugs with an expected treatment duration of >3 months to allow collection of longitudinal data, and had an estimated life expectancy of >6 months. Patients not proficient with any of the available digital patient monitoring (DPM) tool language translations or with psychiatric/neurologic disorders or any condition that might impact the patient’s ability to use the DPM tool were excluded. Patient exclusion and inclusion criteria were in accordance with the locally approved label of the drug used in combination with the treatment-tailored DPM tool.

**Data Collection and Analysis – Time to Healthcare Professional (HCP) Review for Non-alerted vs. Alerted Symptom Questionnaires**

Data extracted from the DPM platform were used to assess what percentage of patients had a symptom alert triggered and the average time in hours until alerted vs. non-alerted symptom questionnaires were reviewed by HCPs.

**Data Collection and Analysis – Health-related Quality of Life (HRQoL)**

Patient HRQoL was assessed using the QLQ-C30 questionnaire at weeks 0, 6, 12, and 18. HRQoL summary score, HRQoL global health status, and symptom control score were assessed. The HRQoL summary score was calculated as the mean of five functional scale scores and eight symptom scales (reversed, with higher scores representing better functioning) excluding financial difficulties. The symptom control score was calculated as the mean of eight symptom scales (reversed, with higher scores representing fewer symptom-related problems) excluding financial difficulties. A Wilcoxon signed rank test was performed for comparisons if data were non-normally distributed. The resulting P values are reported.

**Supplementary online Table 1.** Symptoms covered per drug treatment by the DPM tool used.

| **Symptom term** | **Drug treatment** | | | | | |
| --- | --- | --- | --- | --- | --- | --- |
|  | Immunotherapy (atezolizumab) NSCLC/SCLC &  Immunotherapy (non-drug-specific) NSCLC/SCLC | Immunotherapy (non-drug- or indication-specific) | Immunotherapy combination treatment (non-drug- or indication-specific) | HER2+-targeted treatment (trastuzumab, pertuzumab, or ado-trastuzumab emtansine) mBC/eBC | Chemotherapy | Targeted therapy (TKI) |
|  |  |  |  |  |  |  |
| Abdominal pain | X |  |  | X |  |  |
| Blurred vision |  | X | X |  |  |  |
| Cough | X | X | X | X | X | X |
| Decreased appetite | X | X | X |  | X | X |
| Diarrhea | X | X | X | X | X | X |
| Dizziness |  | X | X |  |  |  |
| Skin dryness |  |  |  | X |  |  |
| Fatigue | X | X | X | X | X | X |
| Headache | X | X | X | X |  | X |
| Itching | X | X | X |  | X | X |
| Joint pain | X |  | X | X | X | X |
| Stomach pain |  | X | X |  |  | X |
| Chest Pain | X | X | X | X |  | X |
| Nausea | X | X | X | X | X | X |
| Rash | X |  | X | X | X | X |
| Shortness of breath | X | X | X | X | X | X |
| Vomiting | X | X | X | X | X | X |
| Constipation |  | X | X | X | X | X |
| Dry mouth | X |  | X | X |  | X |
| Mouth sores |  |  | X |  | X |  |
| Pain and swelling at injection site |  |  |  | X |  |  |
| Heart palpitations |  |  |  | X |  |  |
| Swelling |  |  | X | X | X |  |
| Anxious | X |  |  | X |  |  |
| Taste changes | X |  |  |  |  |  |
| Numbness & tingling |  |  | X | X | X |  |
| Weight loss | X |  |  |  |  |  |
| Fever | X | X | X | X | X | X |
| High blood pressure (Hypertension) |  |  |  | X |  | X |
| Hemorrhage / Bleeds (blood in urine, nosebleed, blood in cough) / Abnormal bleeding |  | X | X | X |  | X |
| Hand and foot syndrome |  |  |  |  |  | X |
| Limb swelling |  |  |  |  |  | X |

**Supplementary online Figure 1.** HCP monitoring of the dashboard.


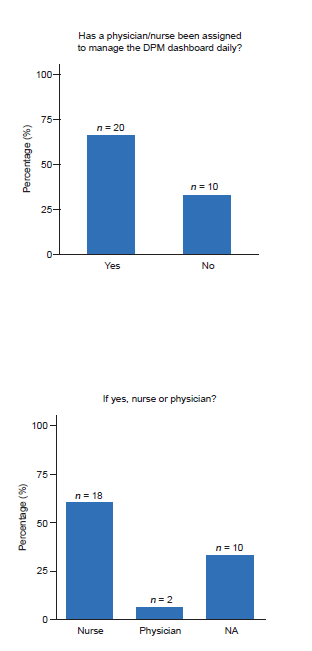


Abbreviations: DPM, digital patient monitoring; HCP, healthcare professional.

**Supplementary online Figure 2.** Time to HCP review for non-alerted vs. alerted symptom questionnaires.

**
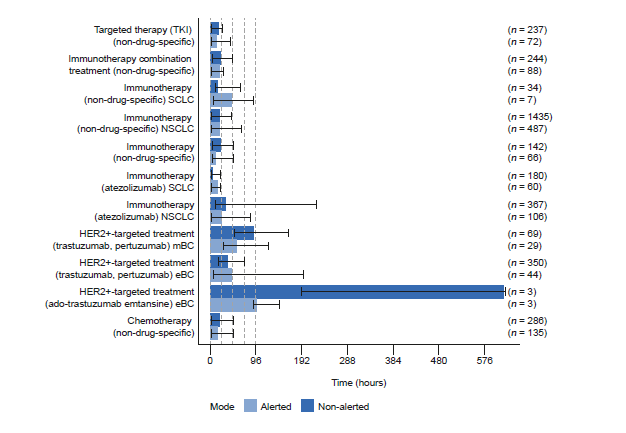
**

Abbreviations: e/mBC, early/metastatic breast cancer; HCP, healthcare professional; NSCLC, non-small cell lung cancer; SCLC, small cell lung cancer; TKI, tyrosine kinase inhibitor.

**Supplementary online Figure 3.** Impact of the DPM tool on HRQoL.

**
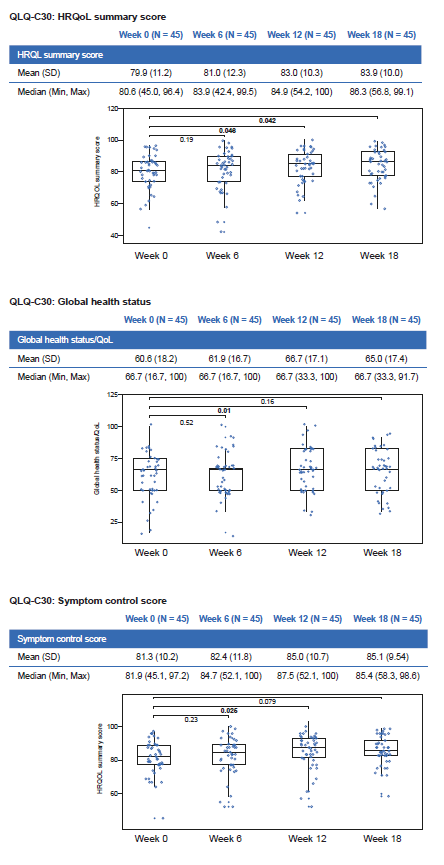
**

Abbreviations: DPM, digital patient monitoring; HRQoL, health-related quality of life;
QLQ-C30, Quality of Life Questionnaire Core 30; SD, standard deviation.
